# What is HOME? Exploring learning themes in a home-visit educational program for postgraduate residents in Taiwan

**DOI:** 10.1007/s41999-025-01283-z

**Published:** 2025-08-02

**Authors:** Yu-Tai Lo, Shih-Ming Li, Chia-Jung Hu, Shih-Han Hsiao, Yi-Ching Yang, Deng-Chi Yang

**Affiliations:** 1 Department of Geriatrics and Gerontology, National Cheng Kung University Hospital, College of Medicine, National Cheng Kung University, Tainan, Taiwan; 2 Department of Public Health, College of Medicine, National Cheng Kung University, Tainan, Taiwan; 3 Department of Geriatric Medicine, School of Medicine, College of Medicine, National Cheng Kung University, Tainan, Taiwan; 4 Department of Counseling and Clinical Psychology, National Dong Hwa University, Hualien, Taiwan; 5 Department of Nursing, Kaohsiung Medical University, Kaohsiung, Taiwan; 6 Institute of Gerontology, College of Medicine, National Cheng Kung University, Tainan, Taiwan; 7 Department of Family Medicine, School of Medicine, College of Medicine, National Cheng Kung University, Tainan, Taiwan; 8 Institute of Clinical Pharmacy and Pharmaceutical Sciences, College of Medicine, National Cheng Kung University, Tainan, Taiwan

**Keywords:** Geriatrics, Home-visit, Experiential learning, Postgraduate medical education, Reflective writing

## Abstract

**Purpose:**

Physician home visits are essential for delivering care to frail, homebound older adults with limited access to routine healthcare. This study examined the experiential learning themes of a home-visit course within a postgraduate year training program in Taiwan.

**Methods:**

A descriptive, exploratory qualitative study was conducted to examine the learning experiences of second-year postgraduate residents who participated in 10 half-days home-visit program as part of their mandatory geriatric medicine training at a university hospital between August 1, 2020, and July 31, 2021. Reflective essays from 47 residents were analyzed using qualitative content analysis to identify key themes.

**Results:**

Three major themes emerged: learning process, learning content, and challenges. The learning process comprised four stages: adaptation, observation, awareness, and reflection. Learning content included a deeper understanding of patients as individuals within the community (holistic care), improved skills in coordinating interdisciplinary teams and integrating patient care information (organizational thinking), greater awareness and empathy through relational practices (mindful engagement), and a stronger appreciation for empowering patients and families through education, goal setting, and community support (empowerment)—conceptualized as the HOME model. Residents also identified challenges in negotiating family dynamics, navigating structural constraints, and reconciling gaps between policy ideals and the realities of home-based care.

**Conclusion:**

These findings support the integration of structured home-visit experiences into postgraduate training to promote competency development, person-centered care, and reflective learning in real-world settings.

**Supplementary Information:**

The online version contains supplementary material available at 10.1007/s41999-025-01283-z.

## Key Summary Points

**Aim:**

We aimed to examine the experiential learning themes of 10 half-days home-visit curriculum for the second-year postgraduate residents using qualitative content analysis of their reflection notes.

**Findings:**

Three major learning themes were identified: (1) a four-stage learning process involving adaptation, observation, awareness, and reflection; (2) learning content related to holistic care, organizational thinking, mindful engagement, and empowerment (HOME); and (3) challenges related to negotiating family dynamics, navigating structural barriers, and reconciling the gap between policy ideals and the realities of home-based care.

**Message:**

Incorporating a home-visit program into medical training could foster meaningful professional growth among postgraduate residents by supporting staged experiential learning, developing core competencies in geriatric care, and encouraging critical reflection on real-world healthcare delivery.

**Supplementary Information:**

The online version contains supplementary material available at 10.1007/s41999-025-01283-z.

## Introduction

Physician home visits are a critical care model for frail, homebound older adults who face substantial barriers in accessing the conventional healthcare settings [1–3]. Although once a cornerstone of clinical practice, home visits have declined with the rise of hospital-based and technology-driven medicine [4, 5]. Nevertheless, the home setting presents unique opportunities for medical trainees to observe patients' lived environments and develop competencies essential to geriatric care, such as patient care, medical knowledge, interpersonal and communication skills, professionalism, systems-based practice, and practice-based learning and improvement [6]. Despite this potential, most residency programs still lack structured training in home-based care [7–9].

This gap is especially concerning given the accelerating global trend of population aging. Taiwan, for instance, became a super-aged society in 2025, with over 20% of its population aged 65 or older [10]. In response, the Taiwanese postgraduate year 2 (PGY2) residency training has included 1-month mandatory geriatric rotations since 2019; however, structured home-visit experiences remain uncommon within these programs [11]. There is a growing need to reimagine training environments that better prepare physicians to deliver care in real-world, community-based settings. This is particularly important, because homebound older adults often present with multimorbidity, functional decline, and psychosocial complexity [12]. The traditional hospital-based curriculum may fall short in preparing physicians to meet these needs.

Contemporary educational frameworks increasingly support embedding learning in authentic care environments and emphasize experiential learning—a process in which learners actively engage and reflect to derive meaning and professional insight [13–15]. Such settings challenge trainees to navigate not only clinical tasks but also relational and logistical complexities, promoting development across cognitive, affective, and social domains [16–18]. Place-based learning, for example, situates education within real-world care contexts and emphasizes interaction between learners, patients, and the community [13]. More recently, ten Cate and colleagues have conceptualized medical competence as a multilayered construct encompassing canonical knowledge, contextual decision-making, and personalized care [19]. This framework supports the argument that is engaging with authentic experiences—such as home visits—is essential for fostering contextual and personalized competencies, particularly during early postgraduate training [20, 21].

Given the underutilization of home visits in PGY curricula and the limited research exploring their educational value in Asia, this study aimed to explore the experiential learning themes of a structured 10 half-days home-visit course embedded in a 40 half-days mandatory geriatric medicine program, and to understand how home-visit experiences contribute to residents' competency development.

## Methods

### Study design

This is a qualitative descriptive study conducted to explore the learning experience from home visit. As the experiential learning themes of home-visit courses are novel in Taiwan, previous data are lacking, and only four-to-five residents per month joined the PGY geriatric medicine course at the hospital selected for this study. Hence, we adopted a qualitative study design to identify and understand the learning experiences during home visits of PGY2 residents. A qualitative approach was selected, because it enabled participants to express themselves while providing data and focused on gaining as much data as possible from a relatively small sample size [22].

Reflective writing was selected as the primary data generation method. It allows physicians to step back, review their actions, and recognize how their thoughts, feelings, and emotions influence decision-making, clinical reasoning, and professionalism [23]. Through this process, participants were encouraged to reflect on their home-visit experiences and articulate personal learning.

### Study setting and curriculum

This study was conducted at National Cheng Kung University Hospital (NCKUH), an 1193-bed tertiary academic medical center in southern Taiwan with a dedicated geriatric service and an affiliated home-care team. Specifically, the PGY2 geriatric medicine curriculum at NCKUH consists of a mandatory 40 half-days rotation comprising 30 half-days in an inpatient geriatric ward and 10 half-days of home-visit experiences. The home-visit course was designed to promote experiential learning in real-world care settings. Residents participated in supervised visits, observed clinical care in patients’ homes, engaged with interdisciplinary teams, and reflected on their experiences through a required written narrative. Detailed home-visit structure and learning objectives are summarized in Table 1. Background information on Taiwan’s national home-care system and the study hospital’s geriatric services is provided in Online Resources 1 and 2, respectively.

**Table 1:** Postgraduate year 2 geriatric medicine home-visit structure

Component	Description
Learning objectives for home visit	(1) Understand operations of home healthcare
	(2) Experience unique characteristics of home-care delivery
	(3) Develop appropriate attitudes toward home-based care
Structure of home visits	(1) Brief orientation before visits (e.g., patient review, safety)
	(2) Two-to-three patient visits per half-day
	(3) Residents accompanied home-care nurses (and occasionally physicians)
	(4) Support in assessing needs, navigating billing, and community resource connection
Residents’ role	Observational and participatory: shadowing, discussion, communication with patients/families
Post-course requirement	A written reflection of at least 800 words

### Ethics approval

This study was approved by the NCKU Human Research Ethics Committee (NCKU-HREC-E-109-491-2). Considering the study’s retrospective nature and the de-identification of data, the HRCE waived the requirement for written informed consent from the participants.

### Data collection

To understand home-visit experiences, the investigators retrospectively collected residents’ personal written reflections (transcripts) on their home-visit experiences in the previous academic year between August 1, 2020, and July 31, 2021, after ethics approval. The residents were asked to reflect on their overall experience, learning outcomes, patient interactions, and interdisciplinary collaboration. These reflections were guided by general thematic prompts (see Online Resource 3). All transcripts were de-identified prior to analysis, and only anonymized data were used. Materials were securely stored without any identifying information.

### Data analysis

We performed a qualitative content analysis to systematically identify patterns and themes within the reflective narratives [24]. Qualitative content analysis was selected for its suitability in organizing and interpreting textual data with both flexibility and methodological rigor. The initial coding process was inductive, allowing themes to emerge from the data.

Two senior geriatricians and medical lecturers (Yang and Lo) independently reviewed the transcripts carefully, familiarized themselves with the data context, and separated the paragraphs based on their meanings on a word-by-word basis. Each transcript was reviewed by two investigators three times, 5–7 days apart. The investigators extracted and encoded key phrases to generate initial codes. After coding, they compared responses and reached consensus in cases of conceptual differences (intercoder agreement) [25].

Each investigator then independently identified and summarized themes based on the coded responses until no new content emerged. Related content was grouped into broader categories using a consensus approach. The transcripts were reviewed multiple times to ensure the uniqueness and consistency of classification. A third investigator (Li), an education researcher specializing in qualitative study methodology, reviewed and cross-validated all transcripts and contents. Triangulation was achieved through the involvement of both clinician and non-clinician analysts [26]. Throughout the analysis, we acknowledged the interpretive nature of qualitative coding. A brief reflexive discussion was held among the authors to reflect on how their clinical backgrounds and assumptions might influence interpretation. To enhance transparency and conceptual clarity, the final themes were derived through a consensus process, aligning both with the data and the research goals [27].

In the later stages of analysis, the emergent codes were then organized deductively into conceptual categories aligned with the research aims and relevant theoretical frameworks, including experiential learning theory and place-based education. This iterative process enabled us to refine and structure the final themes in a way that reflected both participant narratives and broader educational constructs [26, 27].

## Results

**Fig. 1:**
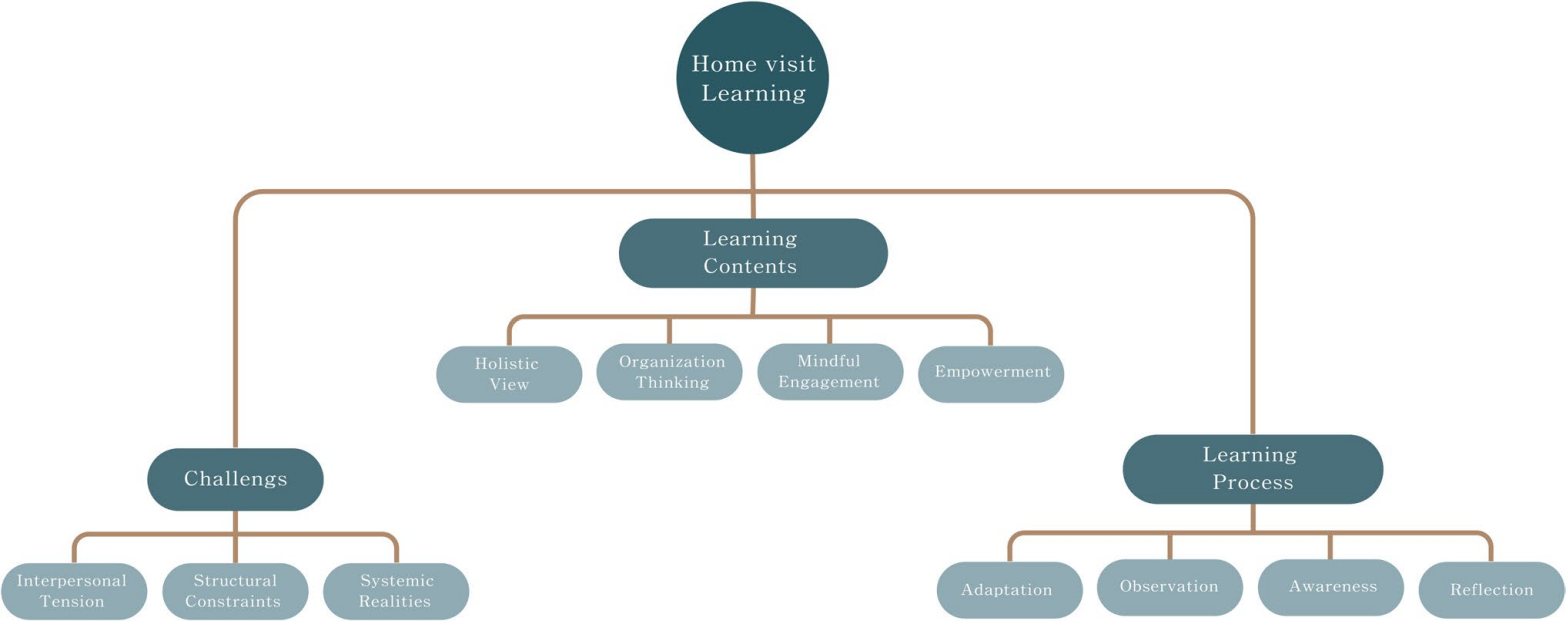
The core themes of home-visit learning

Forty-seven PGY2 residents (mean age 27.2 ± 2.4 years; 57.4% male) completed the training course and submitted their reflection essays during the study period, representing a 100% response rate. Their reflection notes ranged from 800 to 1200 Mandarin words. All were in paragraph format. Following the inductive content analysis, we identified three themes regarding the home-visit experience: learning process, learning contents, and challenges and barriers (Fig. 1).

### The home-visit learning process

Four stages of the learning process were observed from the reflection notes: adaptation, observation, awareness, and reflection.

#### Adaptation

Residents described the need to adjust to a home-care environment along with its unfamiliar challenges, such as limited resources and greater independence. They reported learning to prioritize tasks, solve problems creatively, and become more resourceful in these settings. As one resident explained, “Since I am accustomed to hospitals as the primary setting for medical care, the home environment is undoubtedly new and unfamiliar to me” (PGY2 27).

#### Observation

Through the observation of experienced home-care nurses, residents gained practical insights into patient care and communication strategies. They were particularly struck by the nuanced ways in which nurses built relationships and educated family caregivers. As noted by a resident, “With great attention to detail, the home-care nurse provides comprehensive instructions to the family members and caregivers on patient care, covering topics such as medication use, chest percussion, and feeding” (PGY2 35).

#### Awareness

Residents reported developing greater awareness of care delivery’s broader context, including social, environmental, and psychological factors that influence health outcomes. Further, they became more attuned to the challenges patients and families face at home. As one participant reflected, “Although it was clear that the patient’s daughter was trying her best to follow our instructions, perhaps due to limitations in the caregiver’s physical strength and understanding, the patient’s condition did not seem to improve” (PGY2 7).

#### Reflection

Finally, residents engaged in critical reflection, connecting their experiences with theoretical knowledge and recognizing areas for professional growth. One resident in particular commented, “Home visits allow us to witness a side of medicine that is often overlooked in the hospital setting. While these observations may not be groundbreaking medical discoveries, they help us reconnect with the core of patient care and remind us that fundamental observation and communication are essential for truly understanding our patients” (PGY2 21).

### Key learning contents from home-visit reflections

**Fig. 2:**
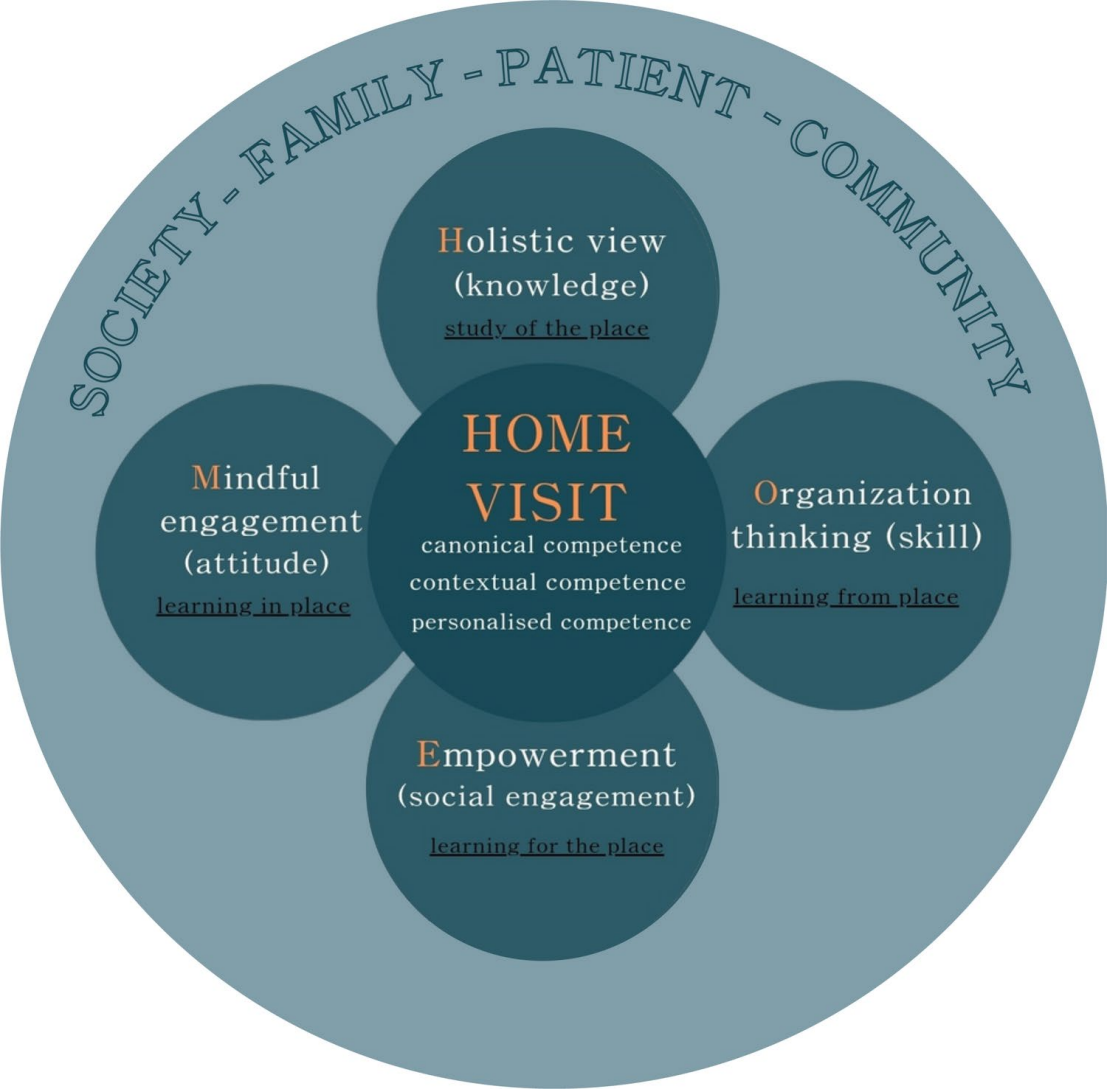
Conceptual model of PGY2 residents’ learning through home visits, aligned with ten Cate’s layered model of medical competence. This figure illustrates how the four experiential learning themes identified in this study—holistic care, organizational thinking, mindful engagement, and empowerment (HOME)—map onto ten Cate’s framework, which describes medical competence as progressing from foundational knowledge and skills to more context-sensitive, personalized, and integrative performance. The model offers a theory-informed lens to interpret how home-visit experiences contribute to the development of person-centered and contextually responsive competencies in postgraduate medical training.

Four subthemes for learning contents-Holistic view, Organizational thinking, Mindful engagement, and Empowerment (HOME)—emerged as recurring components of the residents’ learning experiences during home visits. These themes were derived from qualitative content analysis of reflective narratives and are conceptually illustrated in Fig. 2, which aligns the identified learning domains with ten Cate’s layered model of medical competence.

#### Holistic view

Residents frequently reported that home visits were helpful in broadening their understanding of patients’ lives, shifting their focus from medical conditions to the social and environmental realities that shape health. As one resident reflected, “I realized that the patient's home environment had a significant impact on their recovery. This highlighted the need for a more holistic approach to care that addresses not only the patient's medical needs but also their social and environmental circumstances” (PGY2 10). Another resident noted that “One patient I visited had difficulty preparing meals and keeping their living space clean. I realized these practical issues are just as important as prescribing medication” (PGY2 24). Home visits also helped residents humanize their approach to care, as illustrated by the following comment: “Home visits helped me see beyond the diagnosis. I learned about patients’ life stories, hobbies, and interests, which made my care more personalized and human” (PGY2 27).

#### Organizational thinking

Residents described gaining practical skills, such as organizational thinking in care coordination and interdisciplinary teamwork. One resident noted, “I had to work with the home-care nurse, the pharmacist, and the patient's family to ensure that the medications were delivered as prescribed” (PGY2 14). Others highlighted the importance of communication in aligning care across team members; for instance, one resident explained, “I learned the importance of listening to the patient's concerns and preferences, and then communicating those needs to the rest of the team. This helped to ensure that the patient received the best possible care” (PGY2 42).

#### Mindful engagement

As residents became more present, empathetic, and sensitive to psychosocial dynamics during home visits, many reflections revealed an attitude shift. The PGY must transform their care from hospital to home with mindful engagement. One resident candidly admitted, “When I think back to my time working in the ward, I would frequently judge family members for what I considered to be inadequate care. However, with hindsight, I understand that these caregivers are often under immense pressure and may be struggling with their own health concerns” (PGY2 4). Others emphasized the value of active listening and clear communication, as one participant stated, “During my home visits, I learned the importance of really listening to the patient's stories and concerns” (PGY2 33). Another shared, “I learned that by taking the time to get to know them as individuals, I could provide more compassionate and effective care” (PGY2 40).

#### Empowerment

Home visits also deepened residents’ appreciation for the importance of empowering patients and families in their care journey. One resident noted, “I learned that it's not enough to just provide medical treatment. It's important to empower patients to take an active role in their own care” (PGY2 26). Another emphasized the collaborative aspect of empowerment, stating “By involving the patient's family and community in the care plan, we can create a strong support system that can help the patient cope with their illness and improve their overall quality of life” (PGY2 37).

### Challenges and barriers identified by PGY2 residents

PGY2 residents’ reflective essays revealed a range of challenges and barriers they encountered during their home-visit experiences. Residents identified several key obstacles—such as time constraints, financial disincentives, and limited manpower—that frequently hindered the delivery of quality care. Residents also reflected on deeper challenges related to interpersonal tensions, structure constraints, and the gap between policy ideals and systemic realities in home-based care. These experiences not only affected care delivery but also shaped residents’ professional awareness and reflective learning.

### Negotiating family dynamics and medical authority

Numerous residents described the difficulty of navigating family expectations, particularly when home-visit recommendations conflicted with those of primary care providers. These discrepancies not only challenged their communication skills but also raised questions about professional authority and role clarity. As one resident observed, “Discrepancies between the home-visit physician’s medication adjustments and those prescribed by the primary care provider can lead to hesitation, making effective communication crucial” (PGY2 13). Another resident similarly reflected, “Despite our best efforts, home care can be hindered by factors such as family members’ reluctance to follow care plans and the challenges of coordinating care across different providers” (PGY2 14).

### Navigating structural constraints in home-based care

Frequently, residents noted systemic issues that constrained the delivery of home-based services. These included financial disincentives, time pressures, and insufficient staffing. As one participant explained, “Financially, home visits are often less rewarding, as the income generated is substantially lower than that of clinic visits” (PGY2 30). Another resident remarked, “Home visits often involve a disproportionate amount of time spent per patient, leading to a lower overall patient volume” (PGY2 31). Workforce limitations were also highlighted, with one resident noting, “The shortage of healthcare professionals willing to conduct home visits…present substantial obstacles” (PGY2 38).

### Reconciling idealism with systemic realities

Some residents expressed internal conflict between the ideals of home-based care and the practical limitations they witnessed. They questioned whether home visits could adequately meet complex medical needs and recognized how systemic or cultural pressures often led families to opt for institutional care. One resident reflected, “Despite the family’s good intentions, inadequate care led to complications and hospital readmissions, making me question whether institutional care might have been safer” (PGY2 1). Another added, “Accessible emergency services make it easy for families to rely on hospitals rather than commit to home-based care” (PGY2 38).

## Discussion

This qualitative study explored the reflective writings of PGY2 residents who participated in a structured home-visit course within a postgraduate geriatric medicine training program in Taiwan. Three overarching themes emerged: a progressive learning process, key competencies conceptualized as the HOME model, and barriers and challenges encountered in home-based care. These findings illustrate how home-visit experiences support both competency development and person-centered learning, while also exposing trainees to systemic and interpersonal challenges inherent in real-world geriatric care.

Our findings align with the existing literature and demonstrate that home-visit experiences can foster learners' holistic understanding of patients, improve communication and interprofessional collaboration skills, and deepen empathy and humanism [16, 18, 20, 28]. The four HOME domains—holistic view, organizational thinking, mindful engagement, and empowerment—add to this literature by providing a structured framework that connects experiential learning content with ten Cate’s layered model of medical competence [19]. Further, this model conceptualizes learning progression from canonical knowledge to contextual adaptability and personalized care. While much of the existing literature focuses on medical students [16–18, 21, 22] and family medicine residents [5, 20], our study provides new insights into the learning processes of PGY2 residents, who are at a critical transitional stage between undergraduate medical education and specialty training. In rapidly aging societies such as Taiwan and other Asian countries, such early exposure to home-based care may be particularly relevant for cultivating future-ready physicians.

Our findings also resonate with existing transformative and experiential learning theories. For instance, Mezirow’s theory emphasizes the role of disorienting dilemmas and critical reflection in reshaping learner perspectives [29], while Kolb’s experiential learning model offers a cyclical framework encompassing concrete experience, reflective observation, abstract conceptualization, and active experimentation [30]. In our study, the learning process—adaptation, observation, awareness, and reflection—mirrors these theoretical constructs. As noted in the previous research [28], confronting unfamiliar and complex real-world care scenarios, such as those encountered during home visits, challenges learners to integrate knowledge with values and attitudes. By embedding home visits in transformative and experiential learning frameworks, medical educators can help students develop not only technical competencies but also the critical thinking, empathy, and adaptability necessary to address the complexities of healthcare in a continuously aging society.

In addition to educational benefits, residents' reflections illuminated the challenges and barriers inherent in providing home-based care. These included systemic issues, such as time constraints, financial disincentives, and insufficient manpower, as well as interpersonal challenges in negotiating family expectations and team coordination. Rather than detracting from the learning process, these barriers appeared to enhance it by exposing learners to authentic dilemmas and uncertainty. This aligns with prior findings, suggesting that transformative learning often arises through tension and complexity [20, 29, 31, 32]. By grappling with these real-world complexities, residents moved beyond rote knowledge and procedural tasks toward a more nuanced, context-sensitive understanding of patient care. In this way, the very barriers encountered during home visits served as catalysts for professional growth and the cultivation of reflective practice.

Integration of home visits into geriatrics training programs can be encouraged by embedding structured, longitudinal home-visit experiences in medical and residency curricula, aligning these experiences with core geriatrics competencies, and providing faculty mentorship and interdisciplinary collaboration. Embedding home visits as required components of clerkships or ambulatory rotations has been shown to increase learner interest in geriatrics, improve attitudes toward older adults, and enhance skills in functional assessment, medication management, and understanding of social determinants of health [17, 18, 20, 33–35]. Successful models include pairing trainees with community-dwelling older adults for multiple visits, integrating reflective assignments, and facilitating small-group discussions led by geriatrics faculty. Curricula that use frameworks such as the Geriatrics 5Ms (Mobility, Mind, Medications, Multicomplexity, Matters Most) help structure learning objectives and reinforce key competencies [17, 33]. Interdisciplinary approaches, involving nursing, social work, and pharmacy, further enrich the curricula’s educational value and mirror real-world geriatrics practice [36, 37]. Longitudinal exposure, rather than single visits, is associated with greater improvements in learner confidence and likelihood of incorporating home visits into future practice [20, 33, 38].

Our finding also generated insights that may inform future improvements in curriculum design. For example, incorporating a brief pre-visit orientation or assigning residents to specific roles—such as leading the patient interview or coordinating interprofessional input—may foster more structured learning and reflective engagement [39, 40]. While the home-visit course successfully promoted experiential learning and reflective practice, the residents’ roles during the visits were primarily observational and participatory, guided closely by home-care nurses and, at times, attending physicians. Given that PGY2 residents are in the early stages of clinical training, particularly in geriatric and home-based care, this role structure was intentionally chosen to foster foundational learning through supervised exposure. Nevertheless, future iterations of the curriculum could consider incorporating more clearly defined responsibilities as a means of promoting leadership development and enhancing role clarity. In addition, integrating elements of a focused geriatric assessment—such as evaluating cognition, mood, multimorbidity, and medication use—could help trainees better understand the complexity and goals of home-based care [33, 41]. However, the feasibility of conducting a comprehensive geriatric assessment (CGA) within the limited time frame of a home visit should also be considered when designing such educational components.

Several limitations should be acknowledged when interpreting the findings. First, it was conducted at a single site, the NCKUH, which is an urban tertiary university hospital in Tainan, Taiwan. Hence, the findings might not be generalizable to other academic settings or healthcare systems, particularly to those in different cultural or geographical contexts. Nevertheless, we observed similar learning outcomes to those reported in North America [16–18, 21, 22], and South Africa [28]. Future research should replicate this study across multiple sites, including both urban and rural areas, to enhance its generalizability. Second, the study relied on reflective notes written by PGY2 residents to capture their learning experiences. While this format encouraged authentic self-reflection, it may also be subject to social desirability bias, especially in an educational context where reflection is a graded requirement. Third, we did not evaluate whether residents’ demographic characteristics (e.g., sex or age) influenced the content or tone of their reflections. Future research could address these limitations by employing mixed-methods approaches, developing validated reflection prompts, and incorporating pre–post assessments to better understand learning trajectories. Fourth, the COVID-19 pandemic impacted the study period, particularly toward the end. Some participants had to conduct home visits under pandemic-related restrictions (e.g., wearing personal protective equipment), which could have hindered their communication with patients and their families. This unique context might have influenced the participants’ experiences and reflections differently from those who conducted visits under normal circumstances. Finally, there was variability in the experiences of home visits. Some participants were accompanied by attending physicians and home-care nurses, whereas home-care nurses only accompanied others. While the variability in experiences may limit the specificity of learning themes, the richness and saturation of the qualitative data support the trustworthiness and relevance of the identified themes across contexts.

## Conclusions

This study uncovered valuable insights into the educational benefits of integrating home-visit course into PGY medical training programs in Taiwan. While several challenges were noted, these difficulties may themselves serve as important learning opportunities. The challenges faced during home visits prompted residents to reflect more deeply on communication, interprofessional collaboration, and systems-based practice. As global populations continue to age, incorporating home-visit experiences into postgraduate training may help cultivate more holistic, empathetic, and adaptable clinicians. Our findings suggest that such programs not only enrich early professional development through transformative and experiential learning but also prepare future clinicians to better address the complex needs of aging populations in community-based settings.

## Supplementary Information

Below is the link to the electronic supplementary material.

Supplementary file1 (DOCX 14 KB)

Supplementary file2 (DOCX 14 KB)

Supplementary file3 (DOCX 14 KB)

## Data Availability

Transformed and encrypted individual data of the current study are available from the corresponding author upon the reasonable request.
