# Supplementary material for: What is HOME? Exploring learning themes in a home-visit educational program for postgraduate residents in Taiwan
Source: Eur Geriatr Med. 2025 Aug 2;16(6):2305–14. doi: 10.1007/s41999-025-01283-z (PMC12743701; doi:10.1007/s41999-025-01283-z)
Supplement: Supplementary file 1 — Supplementary file1 (DOCX 14 KB) [file 41999_2025_1283_MOESM1_ESM.docx]

**Online Resources 1. Home care services in Taiwan**

To address the complex needs of homebound older adults, Taiwan’s National Health Insurance (NHI) Administration launched the “Integrated Home-based Medical Care” program in 2016. Under this program, patients are eligible for home visits if they score below 60 on the Barthel Index, experience significant ambulatory difficulty, and are chair- or bed-bound for more than 50% of the day. The services were reimbursed by the National Health Insurance (NHI), although patients were responsible for a 5% co-payment and for covering the transportation costs associated with healthcare providers’ travel to and from the patient’s home. However, the NHI reimbursement guidelines for home medical services stipulate that nurses can conduct home visits at most once every two weeks, and physicians generally conduct such visits once every 2-3 months.
