# Supplementary material for: What is HOME? Exploring learning themes in a home-visit educational program for postgraduate residents in Taiwan
Source: Eur Geriatr Med. 2025 Aug 2;16(6):2305–14. doi: 10.1007/s41999-025-01283-z (PMC12743701; doi:10.1007/s41999-025-01283-z)
Supplement: Supplementary file 2 — Supplementary file2 (DOCX 14 KB) [file 41999_2025_1283_MOESM2_ESM.docx]

**Online Resources 2. Geriatric services in the study hospital**

At the time of the study, National Cheng Kung University Hospital's geriatric services included eight full-time geriatricians and a dedicated 22-bed geriatric ward. Its geriatric outpatient services provide care for over 1,200 patient visits per month, with more than 300 inpatient admissions annually. Additionally, NCKUH operates a home care service department staffed by eight home care nurses, who deliver more than 400 home visits per month. These home visit services are provided within a 10-kilometer radius of the hospital-a range that encompasses both urban and suburban areas, though predominantly serving the urban districts. The home care nurses collaborate with attending physicians from the departments of geriatrics, family medicine, internal medicine, and neurology to provide comprehensive home-based care services.
