# Supplementary material for: What is HOME? Exploring learning themes in a home-visit educational program for postgraduate residents in Taiwan
Source: Eur Geriatr Med. 2025 Aug 2;16(6):2305–14. doi: 10.1007/s41999-025-01283-z (PMC12743701; doi:10.1007/s41999-025-01283-z)
Supplement: Supplementary file 3 — Supplementary file3 (DOCX 14 KB) [file 41999_2025_1283_MOESM3_ESM.docx]

**Online Resource 3. Reflection Prompts**

Residents were asked to submit a written reflection of at least 800 words following their completion of the 10 half-days home visit curriculum. To guide their reflections, the following prompts were provided:

What was your overall impression of the home visit experience?

What were the most memorable or challenging aspects of the home visits?

What did you learn about yourself as a clinician through this experience?

How did your understanding of geriatric care change as a result of this experience?

Describe your interactions with the patient and their family members.

What were the most rewarding or difficult aspects of these interactions?

How did you collaborate with the home care nurse and other members of the healthcare team?

What challenges or benefits did you encounter during the home visits?
